# The General Medical Council

**Published:** 1894-06-02

**Authors:** 


### The Institutional Workshop.

### THE GENERAL MEDICAL COUNCIL.

The General Medical Council assembled for its fifty-sixth session on Tuesday, May 22nd, and terminated its proceedings on Monday, the 28th.

On the first day, after the reception of a new member, and other merely formal proceedings, the President, Sir Richard Quain, Bart., delivered the customary address descriptive of the business awaiting despatch, and the address was ordered to be printed and placed upon the minutes. The sanction of the Council was given to a proposed change in the tenure of office of the assistant examiners in surgery of the Society of Apothecaries, who are appointed by the Council in order to enable the Society to confer a complete medical and surgical qualification. A report was received from the solicitor to the Council, stating that one Joseph Steel, who had been convicted by magistrates for the use of the letters M.D. (Bc.) after his name, letters said to denote a title conferred upon him by a limited company, registered under the Companies' Act by the designation of the "General Council of Safe Medicine," and which implied the possession of a medical qualification, had appealed against his conviction, and that it had been upheld, on which a resolution was passed authorising the President, in conjunction with the legal advisers of the Council, to take such steps as might be advisable to put an end to the granting of so-called degrees by the "General Council of Safe Medicine." A discussion of considerable length arose out of a complaint made by certain practitioners and associations, to the effect that the certificates granted to so-called "midwives," by the Obstetrical Society of London, by the authorities of Queen Charlotte's and of other Lying-in Hospitals, and by certain teachers and lecturers, were of such a character as to be colourable imitations of legal diplomas, and were calculated to impose upon ignorant people, and to lead them to believe that the holders possessed some form of medical qualification. It was ultimately resolved, on the motion of Sir William Turner, seconded by Mr. Brudenell Carter:—

> That, in the opinion of the Council, the issue to women of any certificate of competency, or other document, so framed as to bear a colourable resemblance, either in appearance or phraseology, to a diploma conveying a right to be registered as a medical practitioner, is a proceeding in direct contravention of the spirit of the Medical Act of 1886, and one which will be liable to be visited with the condemnation of the Council. The President is therefore requested to repeat a warning already given, and to urge on the registered practitioners connected with the institutions granting these certificates to reconsider the terms in which they are framed, and to bring them into harmony with this resolution.

A communication from the Parliamentary Bills Committee of the British Medical Association, suggesting certain amendments in the penal clauses of the Medical Acts, was received and entered upon the Minutes. The case of Mr. Edmund Byron de Bonne Robertson, whose name was removed from the Medical Register in May, 1892, was then considered *in camerâ*, on a report from the Executive Committee, and, after deliberation, the name was ordered to be restored.

Wednesday, the 23rd, was given up to the consideration of charges of misconduct, with the result that the names of Mr. George Davidson, Mr. John Melvin Campbell, Mrs. Eliza Foster MacDonogh-Frickhart, Mr. Denis Collins, and Mr. Neville Holland were ordered to be erased from the Medical Register for "infamous conduct in a professional respect." On Thursday other charges were proceeded with, with the result that the name of Mr. Stephen Berry Niblett was erased for "infamous conduct in a professional respect." Mr. Alexander Nairne, against whom a charge had been brought, was not adjudged to have committed more than an indiscretion, and the case of Mr. Robert Ingram Robertson was adjourned in consequence of his absence on account of illness. Resolutions were passed declining to reconsider the case of Mr. E. W. Alabone, or the removal from the Register of certain qualifications formerly possessed by Dr. Herbert Tibbits, but of which he had been deprived by the bodies granting them, and also directing that the general question of the conduct of Dr. Tibbits should be brought before the Council at its next session. Certain matters of detail, with regard to the registration of medical students, were referred to the Education Committee for report.

On Friday, the Council considered reports from its own Pharmacopœia, Finance, Education, and Examination Committees, and resolved, on the motion of Dr. Glover, seconded by Sir Philip Smyly, that it should be "referred to the Executive Committee to obtain legal advice as to what means can be adopted by the Council to stop the practice of persons continuing to use titles attached to diplomas of which they have been deprived, and which no longer appear on the registers." Certain of the standing committees were elected for the ensuing year.

On Saturday, the charge against Mr. Robert Ingram Robertson was proceeded with in his absence, his written defence having been received. He was adjudged to have been guilty of "infamous conduct in a professional respect," and his name was ordered to be erased from the register. In nearly all the penal cases the offence committed was what is technically called "covering" an unqualified person in such a manner as to assist him to evade the law. It was resolved that a warning should be given to the Royal College of Surgeons in Ireland to the effect that the course of study prescribed for its licence in dentistry fell short of the requirements of the Council, and that, unless this course of study were amended, the Council would feel it a duty to report the facts to the Privy Council, in order that the dental licence of the college might no longer be registrable. A long discussion was then held upon the examinations of the Conjoint Board formed in Ireland by the Royal College of Surgeons of Ireland and the Apothecaries Hall of Dublin, and was adjourned until Monday, when resolutions were carried—to the effect: (1) That the final examinations of the Board referred to were insufficient; but (2) that, in consequence of an undertaking to amend, made by the representatives of the licensing bodies concerned, and subject to legal opinion as to the power of the Council to defer reporting insufficiency to the Privy Council, such report should not be made on this occasion, but that all examinations held by the Board during the next twelve months should be specially visited and reported upon. Alterations were' then made in the Standing Orders governing procedure in penal cases, of such a kind as to enable the Council, after having decided that a person summoned before it had committed the offence charged against him, to adjourn the case, and to receive further evidence upon it on a future occasion. The effect of this will be to enable the Council to deal with comparatively venial offences in a manner analogous to that by which, in courts of law, a person may be ordered to come up for judgment when called upon, and also to receive, subsequently to the first hearing, evidence that the course of conduct complained of had been abandoned. Some formal matters were disposed of, and it was then resolved, on the motion of Mr. Brudenell Carter, seconded by Sir Dyce Duckworth, "That the attention of the Council having been called to the practice of advertising by certain dentists, it is hereby resolved that the issue of advertisements of an objectionable character, and especially of such as contain either claims of superiority over other practitioners, or depreciation of them, may easily be carried so far as to constitute infamous or disgraceful conduct in a professional respect." The effect of this warning, coupled with the above described alteration in the Standing Orders in penal cases, will be to enable the Council to hear any case of alleged improper advertising by a dentist, and, if it be proved against him, to give him time to abandon the practice before pronouncing a sentence of removal from the dentists' register. Mr. Carter illustrated the debate on his motion by exhibiting a ghastly collection of pictorial advertisements, most of which had been issued by dentists practising in provincial towns. With this the session was brought to an end, and the Council rose until November.

